# *Agrobacterium rhizogenes* mediated hairy root induction in endangered *Berberis aristata* DC.

**DOI:** 10.1186/s40064-015-1222-1

**Published:** 2015-08-22

**Authors:** Latika Brijwal, Sushma Tamta

**Affiliations:** Department of Biotechnology, Plant Tissue Culture and Molecular Biology Laboratory, Bhimtal Campus, Kumaun University, Nainital, 263136 Uttarakhand India; Department of Botany, Plant Tissue Culture Laboratory, D.S.B.Campus, Kumaun University, Nainital, 263002 Uttarakhand India

**Keywords:** Acetosyringone, Agrobacterium, Berberin, Callus, Hairy root, Transformation

## Abstract

An efficient protocol for hairy root induction in *Berberis aristata* DC. was established using two different strains of *Agrobacterium rhizogenes,* MTCC 532 and 2364 from IMTECH (Institute of Microbial Technology), Chandigarh, India. The strain 532 was more effective than strain 2364 in hairy root induction and in vitro grown callus (61.11 ± 1.60 % transformation frequency) was found to be suitable explant in comparison to leaves (42.59 ± 0.92 % transformation frequency) and nodal segments (34.25 ± 0.92 % transformation frequency) of in vitro grown microshoots for hairy root induction. The presence of *rol* A and *rol B* genes during amplification confirmed the transgenic nature of hairy roots and transformed callus. Transformation frequency of callus was further enhanced (from 61.11 ± 1.60 % to 72.22 ± 1.60 %; when infection time was 1 h) by using acetosyringone (100 µM) during co-cultivation period (48 h) on semisolid MS (Murashige and Skoog) medium. In conclusion, this study describes the protocol for hairy root induction which could further be useful for the production of berberin and may reduce the overharvesting of this endangered species from its natural habitat.

**Electronic supplementary material:**

The online version of this article (doi:10.1186/s40064-015-1222-1) contains supplementary material, which is available to authorized users.

## Background

Tissue culture combined with genetic engineering specifically transformation technology cause improvement and opened new avenues for high volume production of pharmaceutical substances (Hansen and Wright 1999). In this context, induction of hairy root cultures would enhance the production of secondary metabolites. The key characteristic of hairy root culture is its ability to grow rapidly in the absence of exogenous plant growth regulators and hairy roots possess the ability to produce the same compounds found in the parental plants so it can be used as transgenic tool for production of secondary metabolites and recombinant proteins (Kim et al. 2002).

*Berberis aristata* DC. (family Berberidaceae, order Ranunculales) commonly known as ‘Kilmora’ ‘Daruhaldi’ or ‘Indian Berberry’ is a promising source of various secondary metabolites including benzylisoquinoline (berberin), natural alkaloid which is a principle active compound of this plant. Higher amount of berberin is present in plant’s root part in comparison to bark (Andola et al. 2010). *B. aristata* is used in the treatment of diabetes, jaundice, malarial fever; provide relief in eye and ear infection, wound healing, gastrointestinal disorders, effective in rheumatism, shows analgesic action against various skin diseases, anticancer, diuretic, stomachic, and anti-convulsive and also used as a stimulant (Rahman and Ansari 1983; Kirtikar and Basu 1988). In addition to the medicinal properties, it is also used as textile dye, roots used for dyeing cotton cloths and threads and its color can be enhanced by natural mordant (Semwal et al. 2009).

The importance of endangered *B. aristata* (Kala 2002) in different medicine formulations poses an urgent need to develop a protocol which could further be useful for the production of berberin. Therefore keeping the above facts in mind present study has emphasized to develop hairy root induction protocol, which would increase the yield of valuable berberin thereby, reducing the pressure on the endangered plant in its natural population and consequently preventing the overharvesting of *B. aristata* in the native environment.

## Methods

**Fig. 1:**
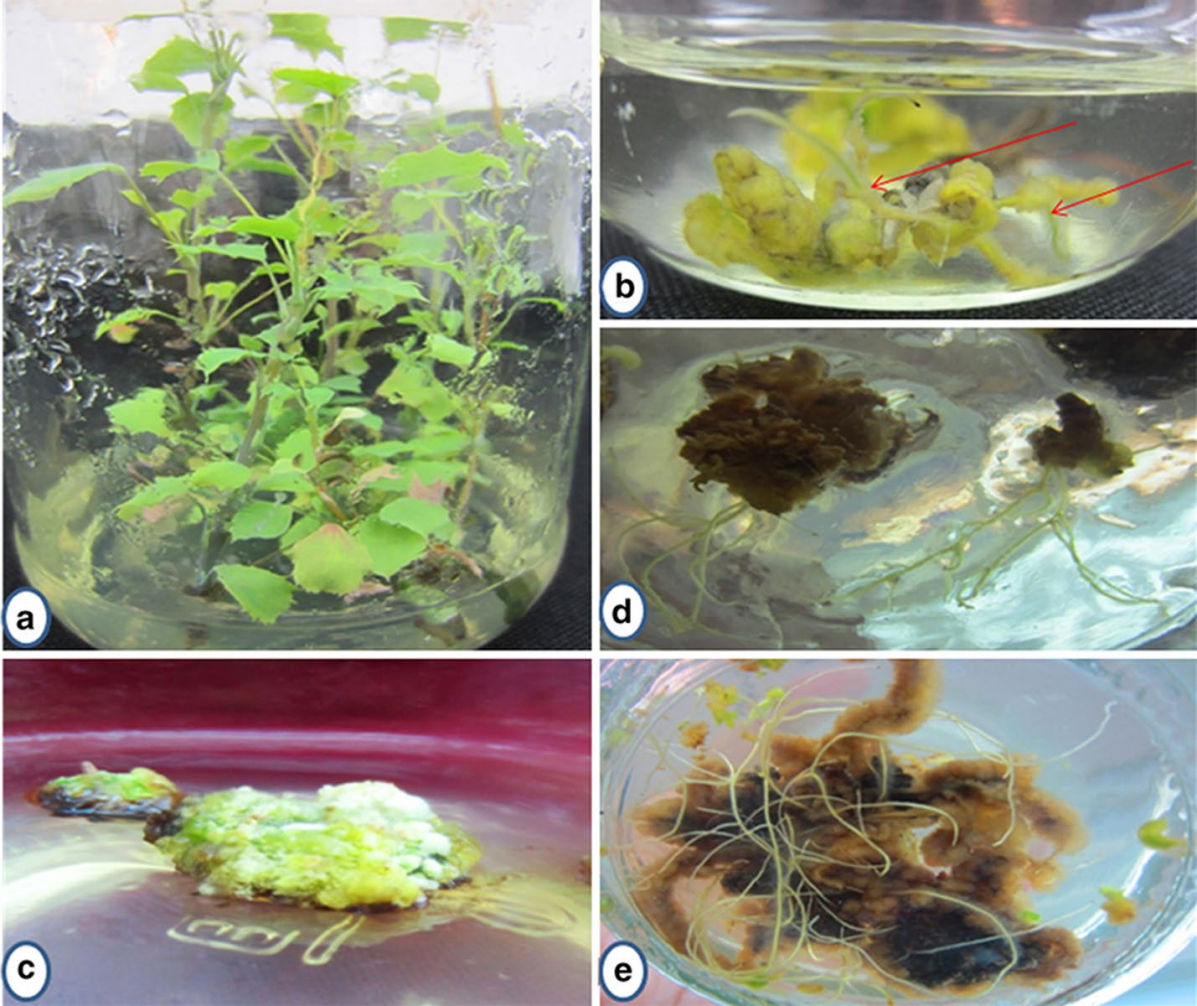
Hairy root induction in *B. aristata* by using *A. rhizogenes* 532 strain. **a** Microshoots used as source of explants for hairy root induction; **b** hairy roots (pointed out by *red colour arrow* in figure) from leaf explant after 28 days; **c** *light green* callus used for hairy root induction; **d**, **e** hairy roots from transformed callus after 20 days of inoculation

In this study three separate experiments were performed. In the first experiment, leaves (approximately 5 × 5 mm size) from in vitro grown microshoots were taken to standardize the infection period and co-cultivation period for hairy root induction. In the second experiment, different explants i.e. leaves (approximately 5 × 5 mm size) and nodal segments (excised from 60 days old in vitro grown microshoots Fig. 1a) and in vitro grown callus (50 days old mature callus; Fig. 1c), were used to select the best one for hairy root induction and in the third experiment, effect of acetosyringone was observed on hairy root induction. In vitro grown microshoots and callus were obtained as described in Brijwal et al. (2015; submitted in In Vitro Cell Dev Biol Plant) and maintained by routine sub culture in their respective media.

### Inoculation of *A. rhizogenes*

*Agrobacterium rhizogenes* strains, MTCC 532 and 2364 purchased from IMTECH (Institute of Microbial Technology), Chandigarh, India and stored in sterile glycerol at −20 °C, were grown in their respective media (100 ml) viz Nutrient Broth and Xanthomonas media at 25 °C under shaking condition (100 rpm) for 48 h. Optical density (O.D) of 48 h old bacterial cultures was determined at 600 nm which was observed to be more than 1.

### Effect of infection and co-cultivation periods on hairy root induction

Leaves from in vitro grown microshoots were taken and cut into small pieces (approximately 5 × 5 mm size), dipped in 48 h old culture of *A. rhizogenes* (MTCC 532 and/or 2364) and pricked before placing in incubator shaker at 25 °C under shaking condition for different time periods (infection time, 15 min to 5 h; Table 1). After that leaves were transferred to PGR-free semisolid MS (Murashige and Skoog) medium (Murashige and Skoog 1962) and placed in culture room at 25 °C ± 2 in dark for 24 or 48 h (Table 1) to check the effect of co-cultivation period. After completion of co-cultivation period the treated leaves were picked and rinsed with double distilled water containing antibiotic (Cephotaxime, 500 mg/l), blotted dry and transferred to semisolid MS medium containing cephotaxime (200 mg/l) and placed again in culture room at 25 °C ± 2 in 16 h light and 8 h dark photoperiod until the roots emerged. Cultures were evaluated on a daily basis.

**Table 1:** Effect of infection and co-cultivation period on hairy root induction in leaves

Infection period	24 h co-cultivation period				48 h co-cultivation period			
	Strain 532		Strain 2364		Strain 532		Strain 2364	
	% transformation frequency	Days required for root induction	% transformation frequency	Days required for root induction	% transformation frequency	Days required for root induction	% transformation frequency	Days required for root induction
0	–	–	–	–	–	–	–	–
15 min	–	–	–	–	–	–	–	–
30 min	–	–	–	–	12.03 ± 0.92^b^	36.66 ± 0.66^e^	–	–
1 h	8.34 ± 0.01^b^	38.66 ± 0.66^e^	–	–	21.46 ± 0.76^c^	35.33 ± 0.33^e^	–	–
2 h	11.11 ± 2.77^b^	35.0 ± 0.57^d^	5.56 ± 2.78^b^	39 ± 0.57^c^	29.62 ± 2.44^d^	32.66 ± 1.45^d^	11.11 ± 2.77^b^	37 ± 0.58^c^
3 h	19.44 ± 2.77^c^	31.33 ± 0.33^c^	11.11 ± 2.77^c^	34.33 ± 0.88^b^	42.59 ± 0.92^e^	27.66 ± 0.33^c^	16.67 ± 0.01^c^	35 ± 0.57^b^
4 h	22.22 ± 2.77^c^	28.33 ± 0.33^b^	–	–	19.44 ± 1.60^c^	25.33 ± 0.33^b^	–	–
5 h	–	–	–	–	–	–	–	–

Values are the mean of % transformation frequency ± SE at p = 0.05, determined by putting mean value of treatments to statistical package SPSS, with one way analysis of variance (ANOVA) and the statistical significance of results was measured using Duncan’s multiple range-Posthoc test. The experiment was performed in triplicates. Value followed by same letter in a column is not significantly different. Data were recorded after 45 days. –: zero transformation frequency

### Effect of different explants on hairy root induction

Different types of explants i.e. leaves (approximately 5 × 5 mm size) and nodal segments (approximately 1.5–2 cm in length) from in vitro grown microshoots and in vitro grown callus (approximately 5 × 5 mm size) were taken, cut into small pieces, dipped in 48 h old culture of *A. rhizogenes* (MTCC 532) and pricked before placing in incubator shaker at 25 °C under shaking condition for different time periods (30 min to 4 h; Table 2). For co-cultivation, infected explants were transferred to PGR-free semisolid MS medium and placed in culture room at 25 °C ± 2 in dark for 48 h (Table 2). Further steps were same as in the above experiment to observe the effect of types of explants on hairy root induction. Cultures were evaluated on a daily basis.

**Table 2:** Effect of different explants on hairy root induction

Infection period (h)	Co-cultivation period (h)	% transformation frequency in		
		Leaf	Nodal segment	Callus
0.0	0.0	–	–	–
0.5	48	12.03 ± 0.92^b^	–	29.03 ± 2.03^b^
1	48	21.46 ± 0.76^c^	–	61.11 ± 1.60^d^
2	48	29.62 ± 2.44^d^	18.55 ± 0.94^c^	36.11 ± 1.60^c^
3	48	42.59 ± 0.92^e^	34.25 ± 0.92^d^	29.62 ± 2.44^b^
4	48	19.44 ± 1.60^c^	10.18 ± 0.92^b^	–

Values are the mean of  % transformation frequency ± SE at p = 0.05, determined by putting mean value of treatments to statistical package SPSS, with one way analysis of variance (ANOVA) and the statistical significance of results was measured using Duncan’s multiple range-posthoc test. The experiment was performed in triplicates. Value followed by same letter in a column is not significantly different. Data were recorded after 45 days. –: zero transformation frequency

### Effect of acetosyringone on hairy root induction

In vitro grown callus and leaves (approximately 5 × 5 mm size) were taken as explants, cut into small pieces, dipped in 48 h old culture of *A. rhizogenes* (MTCC 532) and pricked before placing in incubator shaker at 25 °C under shaking condition for different time periods (1–2 h). Then co-cultivated on PGR-free semisolid MS medium containing acetosyringone (50–150 µM) and placed in culture room at 25 °C ± 2 in dark for 48 h. Further steps were same as in the above experiment to observe the effect of acetosyringone on hairy root induction. In this experiment initially different concentrations of acetosyringone (50–150 µM) were used in co-cultivation period and medium containing 100 µM acetosyringone was found most effective for induction of hairy roots (data was not shown), therefore 100 µM acetosyringone was used to check its effect while using callus or leaf as explant with 1 or 2 h of infection period along with 48 h of co-cultivation period (Table 3). Cultures were evaluated on a daily basis.

**Table 3:** Effect of acetosyringone (100 µM) on hairy root induction

Explant	Infection period (h)	Co-cultivation with acetosyringone (48 h)		Co-cultivation without acetosyringone (48 h)	
		% transformation frequency	Days required for root induction	% transformation frequency	Days required for root induction
Leaf	0	–	–	–	–
	1	30.55 ± 1.60^b^	28.66 ± 0.51^d^	21.46 ± 0.76^b^	27.99 ± 0.38^b^
	2	62.96 ± 2.45^d^	26.22 ± 0.22^c^	29.62 ± 2.44^c^	27.66 ± 0.19^b^
Callus	0	–	–	–	–
	1	72.22 ± 1.60^e^	24.33 ± 0.38^b^	61.11 ± 1.60^e^	27.66 ± 0.50^b^
	2	54.63 ± 2.45^c^	25.77 ± 0.39^c^	36.11 ± 1.60^d^	27.66 ± 0.88^b^

Values are the mean of  % transformation frequency ± SE at p = 0.05, determined by putting mean value of treatments to statistical package SPSS, with one way analysis of variance (ANOVA) and the statistical significance of results was measured using Duncan’s multiple range-posthoc test. The experiment was performed in triplicates. Value followed by same letter in a column is not significantly different. Data were recorded after 35 days. –: zero transformation frequency

### Growth measurement

Hairy root induction could be measured by calculating the hairy root transformation frequency as:

$$\% {\text{ transformation frequency}} = \frac{\text{No of explants inducing hairy roots}}{{{\text{Total no}}.\;{\text{of explants infected with }}A.\;rhizogenes}} \times 100$$

Here only those explants which induced hairy roots were considered as transformed and which did not induce hairy roots were considered as untransformed.

### PCR confirmation of hairy root

PCR was carried out to detect the presence of *rol* gene located on the T-DNA by using a set of *rol* A and *rol* B specific primers (Design by primer3^+^ online software and synthesized by Xcelris labs ltd.; Additional file 1: Table S1). For this genomic DNA was isolated from hairy roots induced from different explants (leaf, nodal segments and callus) and also from transformed callus (which induced hairy roots) by using CTAB extraction method with minor modifications (CTAB extraction buffer 3 ml instead of 5 ml, centrifugation at 12,000 rpm for 15 min instead of centrifugation at 15,000 rpm for 15 min). Genomic DNA from roots of untransformed plants and untransformed callus (which did not induce hairy roots) was used as negative control. Plasmid DNA from bacterial strain was used as positive control. DNA from hairy roots and transformed callus was served as treatments. PCR amplification was carried out in a thermal cycler (Eppendorf, MJ Research PT 200) (Additional file 1: Table S2).

### Data analysis

All the experiments were carried out in triplicate and each treatment contained 9 explants. The results were expressed as mean value ± standard error. The statistical analyses were performed using the statistical package SPSS (Statistical Package for Social Science; version 17). The significance of each group was verified with one-way ANOVA and statistical significance of result measured by using Duncan’s multiple range, Posthoc test (P = 0.05). The graphs were designed using Excel software.

## Results

### Effect of infection and co-cultivation periods on hairy roots induction

Hairy root induction in leaf explant was studied on the basis of percentage transformation frequency and results are given in Table 1 (Additional file 1: Table S3). It was found that changes in infection and co-cultivation periods affected the transformation frequency. Findings of this experiment reflect that when infected leaves were co-cultivated for 24 h, the transformation frequency increased with the increase of infection period up to 4 h for strain 532 and up to 3 h for strain 2364, further increment in infection period caused inhibition of transformation in both the strains. Co-cultivation period of 48 h along with 3 h infection period showed maximum transformation frequency (42.59 ± 0.92 %) after 27.66 ± 0.33 days of inoculation on semisolid MS medium containing cefotaxime (200 mg/l) in case of the strain 532. The strain 2364 showed maximum transformation frequency, (16.67 ± 0.01 %) after 35 ± 0.57 days, when 3 h infection period was used (Table 1). The strain 532 showed better and quick response in comparison to strain 2364. Infection period of 3 h and co-cultivation period of 48 h for leaf explant was found to be the best treatment which was able to show significant frequency of transformation. So 48 h co-cultivation period along with the strain 532 was used in other experiment. Uninfected leaves were not able to show any induction of hairy roots even after 60 days of transfer to semisolid MS medium containing cefotaxime (200 mg/l). Figure 1b shows hairy root formation in leaf explant.

### Effect of different explants on hairy root induction

The effect of different used explants on hairy root induction along with 48 h co-cultivation period for different infection periods (30 min to 4 h) is shown in Table 2 (Additional file 1: Table S4). The variation in type of explants affected the percent transformation frequency. The in vitro grown callus when infected with *A. rhizogene* strain 532 for 1 h and co-cultivated for 48 h on semisolid MS medium showed maximum transformation frequency (61.11 ± 1.60 %; Fig. 1d, e). On the other hand leaf showed only 21.46 ± 0.76 % transformation frequency (Fig. 1b) and shoot did not show any transformation after 1 h infection period treatment. The leaf and nodal segment showed maximum transformation, 42.59 ± 0.92 % and 34.25 ± 0.92 %, respectively, after 3 h infection period. So on the basis of percentage and infection time period, callus was found to be best explant in comparison to nodal segment and leaf (Table 2) for inducing hairy roots on semisolid MS medium.

### Effect of acetosyringone on hairy root induction

Results are presented in Table 3 (Additional file1: Table S5). When 100 µM acetosyringone supplemented in co-cultivation medium, callus attained 72.22 ± 1.60 % transformation frequency after 24.33 ± 0.38 days of inoculation on semisolid MS medium containing cefotaxime (200 mg/l) in comparison to 61.11 ± 1.60 % transformation frequency which was attained in acteosyringone-free co-cultivation medium after 27.66 ± 0.50 days of inoculation. Similar changes were also observed when leaf was used as explant means high transformation frequency was observed when acteosyringone was present in co-cultivation medium (Table 3). The production of hairy roots was enhanced in both callus and leaf explants when co-cultivation medium was supplemented with acetosyringone, which acted as chemotaxis agent. On the basis of the findings of this experiment it was concluded that acetosyringone not only enhanced the transformation rate but also decreases the time period required for root initiation.

### Confirmation of transgenic

**Fig. 2:**
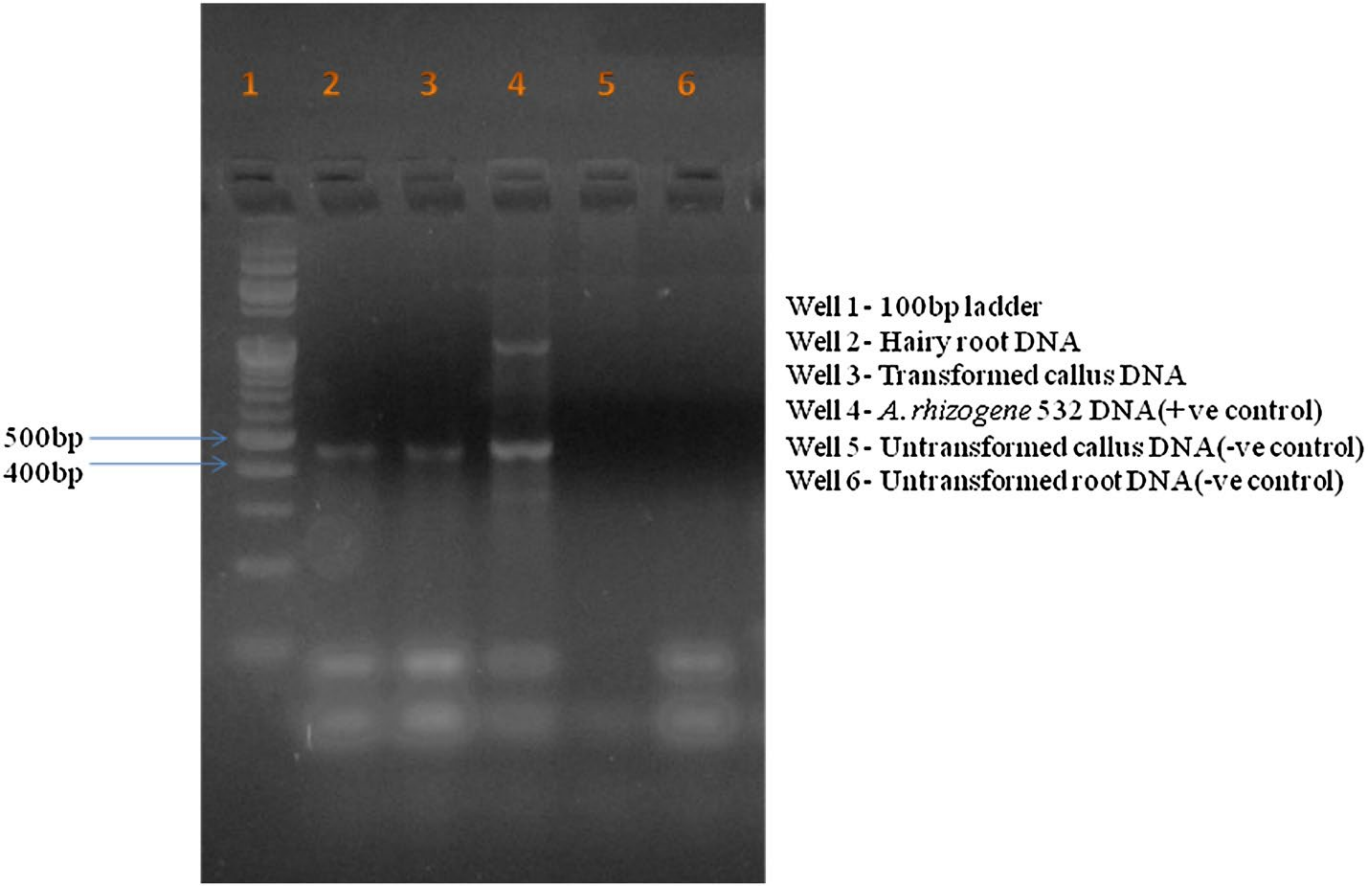
PCR amplification of *rol* A gene

**Fig. 3:**
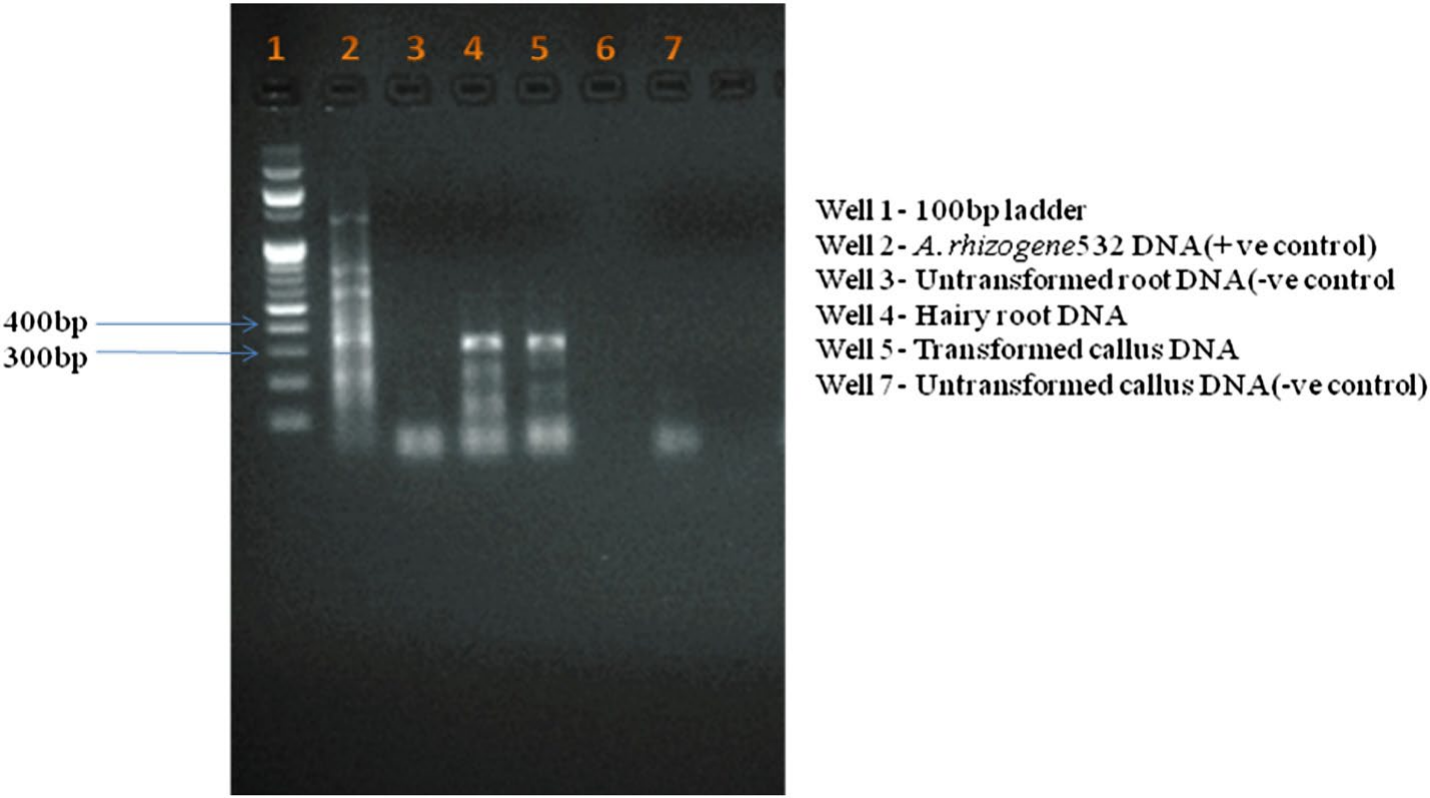
PCR amplification of *rol* *B* gene

The presence of *rol* A and *rol B* genes confirmed the transgenic nature of hairy roots and transformed callus. DNA extracted from hairy roots and transformed callus after PCR amplification demonstrated the product of expected size approximately 500 bp with used *rol A* primer and approximately 300 bp product with used *rol B* primer. They were not observed in PCR product of untransformed root and callus DNA, which were used as negative control. The amplicon size observed from bacterial strain DNA exactly matched with hairy root and transformed callus product (Figs. 2, 3) which conforming the transgenic nature of hairy roots and callus.

## Discussion

Virulency of the *A. rhizogenes* varies according to the strain that affected the frequency of the hairy root production (Giri et al. 2001). There are number of methods for genetically modifying the plants but most common and effective methods is—*Agrobacterium rhizogenes* mediated transformation, also known as “natural genetic engineer”, by inducing proliferative roots at the site of infection because it inserted genetic material (T-DNA) into plant genome by which *rol* gene express. In this experiment two types of strains were used and on the basis of percentage transformation frequency, it was found that MTCC strain 532 (ATCC 1532) was more effective. Strain 532 is also effective in case of *Plumbago rosea*, *Rubia tinctorum*, *Arachis hypogaea* and *Withania somnifera* hairy roots cultures in comparison to other strains (Yogananth and Basu 2009; Ercan and Taskin 1999; Karthikeyan et al. 2007; Doma et al. 2012).

The duration of infection time and co-cultivation period also have an effect on the frequency of hairy roots. During this experiment, maximum percent transformation occurred in strain 532 when explants infected for 3 h in bacterial culture and co-cultivated for 48 h in semisolid MS medium. On the other hand when co-cultivated for 24 h, percent transformation frequency was low (3 h infection period). The data showed that infection period along with co-cultivation period affected hairy root induction. Co-cultivation period of 48 h was also found effective for *Glycyrrhiza glabra*, *Linum mucronatum* and *Artemisia annua* cultures (Mehrotra et al. 2008; Samadi et al. 2012; Giri et al. 2001). Short infection periods (15–30 min) were not effective probably due to insufficient time for bacterial infection.

The types of explants also influenced the hairy roots production. During the experiment, three types of explants were used. Callus showed maximum transformation frequency followed by leaf after 1 h infection period. As explants, nodal segments showed lowest frequency of transformation because stem contains less meristematic activity in comparison to leaves and callus. The transformed callus culture also assumes importance on the basis of secondary metabolites production (Bulgakov et al. 2008).

The mechanism of transformation depends upon the activity of *A. rhizogenes* induced by plant wound’s phenotypic compound such as acetosyringone (Kumar et al. 2006). Acetosyringone enhanced the transformation rate of infected explants by activation of *vir* genes (Gelvin 2000). The findings of this experiment conclude that acetosyringone (100 µM) in co-cultivation medium not only enhanced transformation of callus but also decreased the time required for hairy roots induction in comparison to acetosyringone-free treatment. Similar effects were observed when acetosyringone was used for *loblolly pine*, and *Nicotiana tabaccum* plant (Tang 2003; Kumar et al. 2006).

## Conclusions

In conclusion, this study describes the protocol for hairy root induction which could further be useful for the production of berberin and may reduce the overharvesting of this endangered species from its natural habitat.

## Additional file

Additional file 1. In the Supplemental Material Section primers for *rol* A, *rol* B genes and PCR amplification profile used for PCR confirmation of hairy root as well as ANOVA results for Tables 1, 2 and 3 are presented.
