# Supplementary material for: Agrobacterium rhizogenes mediated hairy root induction in endangered Berberis aristata DC
Source: Springerplus. 2015 Aug 22;4:443. doi: 10.1186/s40064-015-1222-1 (PMC4546071; doi:10.1186/s40064-015-1222-1)
Supplement: Additional file 1. — In the Supplemental Material Section primers for rol A, rol B genes and PCR amplification profile used for PCR confirmation of hairy root as well as ANOVA results for Tables 1, 2 and 3 are presented. [file 40064_2015_1222_MOESM1_ESM.docx]

**Supplementary Table S1: Primers for *rol A* and *rol B* genes**

|  | **Forward primer** | **Reverse primer** |
| --- | --- | --- |
| **For *rol A* gene** | 5ˈ-AAGAAGGCCAAACGTGTGTC-3ˈ | 5ˈ-TGAATATTCCGGTCCAGCGA-3ˈ |
| **For *rol B* gene** | 5ˈ- CAACGCTTTCAACCACGAGA-3ˈ | 5ˈ- TCAGAAGATGGGCCAGTTGT-3ˈ |

**Supplementary Table S2: PCR amplification profile**

| **Cycle NO.** | **Denaturation** | | **Annealing** | | **Extension** | |
| --- | --- | --- | --- | --- | --- | --- |
|  | **Temperature** | **Time** | **Temperature** | **Time** | **Temperature** | **Time** |
| **1^st^ cycle** | 95°C | 5 min. |  |  |  |  |
| **34 cycle** | 95°C | 30 sec. | 53°C rolB | 1 min. | 72°C | 1min. |
|  |  |  | 46°C rolA |  |  |  |
| **35^th^ cycle** |  |  |  |  | 72°C | 5 min. |

**Supplementary Table S3: Effect of infection and co-cultivation period on hairy root induction in leaves**

ANOVA Summary-

| **Source of variation** | **df** | **24 h co-cultivation time** | | | | | | | | **48 h co-cultivation time** | | | | | | | |
| --- | --- | --- | --- | --- | --- | --- | --- | --- | --- | --- | --- | --- | --- | --- | --- | --- | --- |
|  |  | **% transformation frequency in strain 532** | | **Days required for root induction** | | **% transformation frequency in strain 2364** | | **Days required for root induction** | | **% transformation frequency in strain 532** | | **Days required for root induction** | | **% transformation frequency in strain 2364** | | **Days required for root induction** | |
|  |  |  |  |  |  |  |  |  |  |  |  |  |  |  |  |  |  |
|  |  | **Mean Squares** | **F ratio** | **Mean Squares** | **F ratio** | **Mean Squares** | **F ratio** | **Mean Squares** | **F ratio** | **Mean Squares** | **F ratio** | **Mean Squares** | **F ratio** | **Mean Squares** | **F ratio** | **Mean Squares** | **F ratio** |
|  |  |  |  |  |  |  |  |  |  |  |  |  |  |  |  |  |  |
| **Between Groups** | 7 | 256.34 | 29.55 | 978.19 | 2608.5 | 51.31 | 8.86 | 868.95 | 2085.48 | 735.97 | 180.86 | 839.94 | 775.34 | 130.69 | 45.21 | 834 | 3336 |
| **Within Groups** | 16 | 8.67 |  | 0.37 |  | 5.79 |  | 0.41 |  | 4.069 |  | 1.08 |  | 2.89 |  | 0.25 |  |
| **Total** | 23 |  |  |  |  |  |  |  |  |  |  |  |  |  |  |  |  |
| **LSD** |  | 11.15 | | 42.53 | | 2.23 | | 37.78 | | 32 | | 36.52 | | 5.68 | | 36.26 | |

**Supplementary Table S4: Effect of different explants on hairy root induction-** ANOVA Summary-

| **Source of variation** | **% transformation frequency in** | | | | | | |
| --- | --- | --- | --- | --- | --- | --- | --- |
|  | **df** | **Leaf** | | **Nodal segment** | | **Callus** | |
|  |  |  |  |  |  |  |  |
|  |  | **Mean Squares** | **F ratio** | **Mean Squares** | **F ratio** | **Mean Squares** | **F ratio** |
|  |  |  |  |  |  |  |  |
|  |  |  |  |  |  |  |  |
| **Between Groups** | 5 | 638.73 | 117.72 | 576.05 | 443.28 | 1625.7 | 212.66 |
| **Within Groups** | 12 | 5.42 |  | 1.29 |  | 7.64 |  |
| **Total** | 17 |  |  |  |  |  |  |
| **LSD** |  | 37.57 | | 33.89 | | 95.63 | |

**Supplementary Table S5: Effect of acetosyringone (100µM) on hairy root induction-** ANOVA Summary-

| **Source of variation** | **Co-cultivation with acetosyringone**  **(48 h)** | | | | | **Co-cultivation without acetosyringone**  **(48 h)** | | | |
| --- | --- | --- | --- | --- | --- | --- | --- | --- | --- |
|  | **Df** | **% transformation frequency** | | **Days required for root induction** | | **% transformation frequency** | | **Days required for root induction** | |
|  |  |  |  |  |  |  |  |  |  |
|  |  | **Mean Squares** | **F ratio** | **Mean Squares** | **F ratio** | **Mean Squares** | **F ratio** | **Mean Squares** | **F ratio** |
|  |  |  |  |  |  |  |  |  |  |
|  |  |  |  |  |  |  |  |  |  |
| **Between Groups** | 4 | 2539.44 | 246.439 | 420.66 | 1133.265 | 1483.404 | 211.02 | 462.016 | 631.164 |
| **Within Groups** | 10 | 10.305 |  | 0.371 |  | 7.03 |  | 0.732 |  |
| **Total** | 14 |  |  |  |  |  |  |  |  |
| **LSD** |  | 181.39 | | 30.05 | | 105.96 | | 33.00 | |
